# Scrub Typhus Mimicking Severe Community-Acquired Pneumonia: A Diagnostic Challenge

**DOI:** 10.7759/cureus.80660

**Published:** 2025-03-16

**Authors:** Vijay Sundar Singh, Manoj Kumar, Afra Shibin, Monish Thomas, Chrisel Sequeira

**Affiliations:** 1 Critical Care Medicine, Father Muller Medical College, Mangalore, IND; 2 Anesthesiology, Father Muller Medical College, Mangalore, IND; 3 Internal Medicine, Father Muller Medical College, Mangalore, IND

**Keywords:** doxycycline, eschar, pleural effusion, pneumonia, scrub typhus

## Abstract

A 23-year-old previously healthy female presented with a five-day history of high-grade fever, productive cough, throat pain, and progressive breathlessness. On admission, she was in respiratory distress with severe hypoxia requiring high-flow nasal cannula support. Chest x-ray revealed extensive bilateral lower lobe consolidation, moderate left-sided pleural effusion, and right-sided minimal pleural effusion. Initial empirical treatment with ceftriaxone and azithromycin for community-acquired pneumonia failed to improve her condition. High-resolution computed tomography of the chest confirmed multilobar consolidation, prompting further infectious workup. Serology for scrub typhus was positive, leading to a diagnosis of scrub typhus pneumonia. The patient was transitioned to targeted therapy with doxycycline, resulting in rapid clinical improvement. Oxygenation improved, inflammatory markers declined, and she was successfully weaned off non-invasive ventilation. However, she developed persistent hoarseness, and laryngoscopic evaluation revealed post-infectious laryngitis with reduced vocal cord mobility. The condition was managed conservatively with voice rest and steam inhalation, leading to gradual resolution. She was discharged after 14 days with complete respiratory recovery.

Scrub typhus pneumonia typically involves the lower lobes, but multilobar involvement, upper lobe consolidation, and pleural effusion are rare. Delayed diagnosis and the absence of a characteristic eschar can contribute to treatment delays. Early suspicion, serological testing, and initiation of doxycycline are essential for favorable outcomes. Our case highlights the need to consider scrub typhus in patients with severe, atypical pneumonia, particularly in endemic regions.

## Introduction

Scrub typhus, a vector-borne zoonotic disease caused by the obligate intracellular bacterium Orientia tsutsugamushi, remains a significant public health challenge across the Asia-Pacific region, particularly within the endemic area known as the “tsutsugamushi triangle” [1]. A recent study assessing the burden of scrub typhus in India, a country within this geographical hotspot, reported its involvement in at least 25.3% of cases of acute undifferentiated febrile illness (AUFI) [2]. The disease typically follows an incubation period of six to 21 days, presenting with fever, headache, myalgia, and gastrointestinal symptoms [3].

The pathophysiology of scrub typhus is primarily driven by the infection of endothelial cells, leading to vasculitis and subsequent infiltration of T cells and macrophages. This triggers a complex inflammatory cascade, wherein both endothelial and non-endothelial cells produce cytokines that not only contribute to host defense but also induce tissue damage. The resulting immune-mediated injury can lead to severe complications, including hepatitis, renal failure, meningoencephalitis, myocarditis, and acute respiratory distress syndrome (ARDS) [1].

Scrub typhus primarily presents with nonspecific febrile illness, often accompanied by generalized lymphadenopathy, cough, eschar formation, and gastrointestinal symptoms [3]. While pulmonary complications, such as interstitial pneumonia, pulmonary edema, and hemorrhage, have been documented, lobar pneumonia remains a rare presentation of scrub typhus. In severe cases, acute respiratory failure and ARDS may develop, reflecting the extensive endothelial injury and immune dysregulation associated with the disease [4].

This interplay underscores the need for early recognition and timely intervention to prevent life-threatening complications. Here, we report a unique case of scrub typhus presenting as lobar pneumonia in a young female, emphasizing the importance of considering scrub typhus in patients with atypical pneumonia in endemic regions.

## Case presentation

A 23-year-old female with no known comorbidities presented to the emergency department with a five-day history of productive cough, high-grade fever with chills and rigors, and throat pain. She also reported hoarseness of voice for the last two days. Her symptoms had progressively worsened over the past two days, with increasing breathlessness.

On admission, she was alert, oriented, and in respiratory distress. She exhibited increased work of breathing with nasal flaring and the use of accessory muscles. Her heart rate was 119 bpm, blood pressure was 92/64 mmHg, oxygen saturation (SpO₂) was 90% on a Venturi mask (0.6 FiO₂), and respiratory rate was 45 breaths per minute. A respiratory examination revealed coarse crackles and diminished air entry on the left side. As the patient did not show improvement with the Venturi mask, she was initiated on high-flow nasal cannula (HFNC) in the ICU with 60% FiO₂ and a flow rate of 50 L/min.

Arterial blood gas (ABG) analysis revealed severe hypoxia with a P/F ratio of 91. Chest X-ray and lung ultrasound demonstrated a left-sided moderate pleural effusion with underlying collapse-consolidation, along with a right-sided minimal pleural effusion and consolidation. Laboratory investigations showed a normal total leukocyte count (TLC) of 5,220 cells/mm³, C-reactive protein (CRP) of 205 mg/L, and procalcitonin of 0.302 ng/mL. Table 1 shows Laboratory and ABG Investigations at ICU admission. The patient was initially started on empirical treatment for community-acquired pneumonia (CAP) with intravenous ceftriaxone and azithromycin. Due to persistent severe hypoxia and respiratory distress despite HFNC support, the patient was transitioned to non-invasive ventilation (NIV).

**Table 1:** Laboratory and arterial blood gas parameters at admission. CRP - C-Reactive Protein, SGOT - Serum Glutamic-Oxaloacetic Transaminase, SGPT - Serum Glutamic-Pyruvic Transaminase, PaO₂ - Partial Pressure of Oxygen in Arterial Blood, PaCO₂ - Partial Pressure of Carbon Dioxide in Arterial Blood, HCO₃⁻ - Bicarbonate, FiO₂ - Fraction of Inspired Oxygen.

Parameters	At admission	Reference values
Total leukocyte counts (cells/mm^3^)	5,220	4,000 – 10,000
Neutrophil, %	89	48 – 80
Lymphocytes, %	08	20 – 40
Platelet count (cells/mm^3^)	289,000	150,000 – 410,000
Hemoglobin (g/dL)	9.9	12 – 16
CRP (mg/L)	205	0 – 5
Procalcitonin (ng/mL)	0.302	<0.5
Urea (mg/dL)	68	15 – 36
Creatinine (mg/dL)	1.2	0.6 – 1.2
Bilirubin total (mg/dL)	0.46	0.2 – 1.3
Conjugated bilirubin (mg/dL)	0.38	0 – 0.4
Unconjugated bilirubin (mg/dL)	0.08	0.1 – 1
SGOT (IU/L)	151	14 – 36
SGPT (IU/L)	102	0 – 35
Total protein (g/dL)	6.2	6.3 – 8.2
Albumin (g/dL)	2.8	3.5 – 5
Sodium (mEq/L)	127	136 – 145
Potassium (mEq/L)	3.3	3.5 – 5.1
Chloride (mEq/L)	96	98 – 107
pH	7.50	7.35 – 7.45
PaO₂ (mmHg)	55	80 – 100
PaCO₂ (mmHg)	34	35 – 45
HCO₃⁻ (mmol/L)	26.5	21 – 28
Lactate (mmol/L)	1.0	0.5 – 2.2
FiO₂	0.6	0.21 – 1

**Figure 1:**
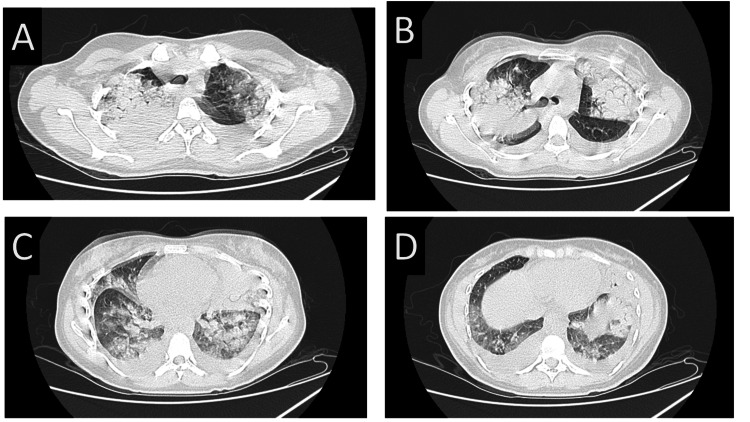
High-resolution CT thorax showing multilobar pneumonia with bilateral consolidations and pleural effusion. (A, B) Bilateral upper lobe consolidation with ground-glass opacities. (C, D) Bilateral lower lobe consolidation, pleural effusion with ground-glass opacities.

An initial diagnostic assessment for CAP was conducted, including blood cultures, sputum Gram stain, sputum culture, and a PCR-based respiratory panel for detecting atypical bacterial and viral pathogens. Since there was no improvement after 48 hours despite broad-spectrum empirical antibiotic therapy and the initial assessment for a causative agent for CAP was negative, a high-resolution computed tomography (HRCT) of the chest was performed, which revealed extensive consolidation in the left lower lobe with air bronchograms, patchy right lower lobe opacities, and bilateral pleural effusions. Figures 1A-1D show the HRCT chest with bilateral lower lobe consolidation with air bronchograms, patchy bilateral lower lobe consolidation with ground glass opacities, and bilateral pleural effusions. Given these findings, pleural fluid drainage was performed. The pleural fluid appeared slightly turbid and no visible blood staining was noted. Analysis of the pleural fluid revealed a TLC of 600/mm³ with a differential count of 40% neutrophils and 60% lymphocytes. The pleural protein level was 3.2 g/dL, lactate dehydrogenase (LDH) was 1,047 IU/L, pH was 7.3 and sugar was 54 mg/dL. Using Light’s criteria, the pleural fluid was classified as an exudative effusion. Pleural cultures showed no growth.

Considering the patient's persistent fever, lack of response to empirical antibiotics, and the presence of pleural effusion with consolidation, an extended infectious workup was undertaken, including serologies and PCR for scrub typhus, leptospirosis, dengue, malaria, and tuberculosis, which revealed scrub typhus IgM positivity. The antibiotic regimen was revised to include intravenous doxycycline along with azithromycin for targeted therapy.

Over the next few days, the patient's oxygenation showed significant improvement, with the P/F ratio rising to above 200. Despite the initial severity of hypoxia, she responded well to supportive care, including NIV. The NIV was continued as her respiratory function gradually improved, and she was closely monitored for any signs of deterioration. Her inflammatory markers showed a downward trend, and fever defervescence was noted by day 3 of targeted therapy. A repeat microbiological assessment confirmed that scrub typhus IgM remained persistently positive, reinforcing the diagnosis and the need for continued doxycycline therapy.

As her respiratory status improved further, she was successfully weaned off NIV in a stepwise manner. She tolerated the discontinuation of NIV well and was transitioned to nasal prongs for oxygen supplementation. Her respiratory effort normalized, and she was able to maintain adequate oxygenation on room air. By day 7, she remained afebrile and hemodynamically normal. With resolution of respiratory distress and sustained improvement, she no longer required respiratory support and was deemed fit for transfer to the general ward by day 10 for further observation and supportive care.

**Figure 2:**
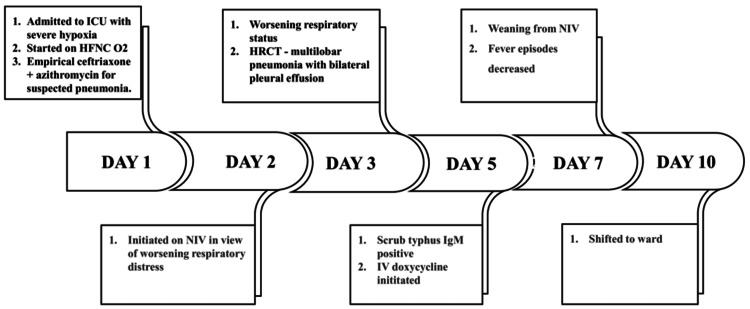
Timeline of events in ICU course of patients' stay. ICU - Intensive Care Unit, HFNC - High-Flow Nasal Cannula, O₂ - Oxygen, HRCT - High-Resolution Computed Tomography, NIV - Non-Invasive Ventilation, IgM - Immunoglobulin M, IV - Intravenous

The patient was monitored in the ward, where she was noted to have persistent hoarseness of voice. A laryngoscopic examination revealed reduced bilateral vocal cord mobility with a mild phonatory gap, suggesting post-infectious laryngitis. She was advised absolute voice rest and steam inhalation. She was discharged on day 14 with instructions for follow-up in the outpatient department. A repeat chest x-ray showed improvement. Figure 2 shows the timeline of events in the ICU course of the patient's stay.

## Discussion

Scrub typhus, caused by Orientia tsutsugamushi, is an acute febrile illness endemic to tropical and subtropical regions. While it predominantly presents as a febrile illness with eschar formation and lymphadenopathy, severe forms can involve multiple organ systems, including the lungs, leading to pneumonia and acute respiratory distress syndrome (ARDS) [1]. This case highlights an unusual presentation of scrub typhus with severe multilobar pneumonia and pleural effusion, which necessitated intensive care support and NIV. The delay in diagnosis due to the atypical presentation underscores the challenges associated with recognizing scrub typhus pneumonia in non-endemic areas or cases lacking classical eschars.

Scrub typhus can lead to pulmonary involvement in 20%-30% of cases, with manifestations ranging from mild interstitial pneumonia to severe ARDS [5]. The primary pathophysiological mechanism involves endothelial damage due to O. tsutsugamushi, leading to increased vascular permeability, capillary leakage, and inflammation [6]. The resultant alveolar injury and interstitial edema contribute to hypoxia and respiratory failure, as observed in this patient.

Multilobar involvement in scrub typhus pneumonia is rare but has been reported in severe cases. In this case, HRCT revealed bilateral lower lobe consolidation with air bronchograms, patchy ground-glass opacities, and bilateral pleural effusions. This extensive lung involvement suggests a more aggressive inflammatory response, which could have been exacerbated by delayed diagnosis and initiation of targeted therapy.

Scrub typhus pneumonia typically involves the lower lobes; however, upper lobe consolidation has been reported in rare cases [7]. The presence of upper lobe consolidation in this patient suggests a more diffuse and aggressive form of the disease, possibly due to increased vascular permeability and severe endothelial inflammation. Additionally, moderate pleural effusion, though uncommon, has been documented in some cases of scrub typhus pneumonia [8]. The pleural effusion in this patient was classified as an exudative effusion, likely secondary to the intense inflammatory response triggered by O. tsutsugamushi infection. These atypical features highlight the need for a broad differential diagnosis in cases of severe pneumonia with pleural effusion, especially in endemic areas.

The initial presentation of this patient with fever, productive cough, and pleural effusion closely mimicked bacterial CAP. The lack of improvement with broad-spectrum antibiotics necessitated an expanded infectious workup, which ultimately led to the diagnosis of scrub typhus. Differentiating scrub typhus pneumonia from bacterial, viral, and other atypical pneumonia is challenging due to overlapping clinical and radiological features. However, a lack of response to empirical antibiotics and persistent fever should raise suspicion for alternative etiologies, including rickettsial infections.

Serological tests, including IgM ELISA, and PCR-based assays play a crucial role in diagnosing scrub typhus, especially in patients without eschar formation [9]. In this case, positive scrub typhus IgM serology confirmed the diagnosis, prompting a switch to add doxycycline with azithromycin, which led to rapid clinical improvement.

Doxycycline remains the first-line treatment for scrub typhus, with rapid defervescence typically observed within 48-72 hours of therapy initiation. In this case, the patient exhibited significant improvement in oxygenation and inflammatory markers after starting doxycycline, underscoring its efficacy in severe pulmonary involvement. While alternative agents like azithromycin and rifampin have been studied, doxycycline remains the preferred choice due to its superior efficacy in severe cases [10].

Supportive care, including oxygen therapy and non-invasive ventilation, played a crucial role in stabilizing the patient. NIV was required due to persistent respiratory distress and severe hypoxia, highlighting the importance of early respiratory support in preventing invasive ventilation.

Post-infectious laryngitis following scrub typhus is an unusual but documented complication. The inflammation triggered by O. tsutsugamushi can extend to the laryngeal mucosa, leading to edema and impaired vocal cord mobility]. In this patient, persistent hoarseness of voice was noted during recovery, and laryngoscopic examination revealed reduced bilateral vocal cord mobility with a mild phonatory gap. The management of post-scrub typhus laryngitis is primarily supportive, including absolute voice rest, steam inhalation, and hydration. In refractory cases, short courses of corticosteroids may be considered to reduce inflammation and expedite recovery [11].

The prognosis of scrub typhus pneumonia depends on the severity of lung involvement and the timeliness of treatment initiation. In this case, despite severe hypoxia with a P/F ratio of 91 at admission, early initiation of doxycycline and supportive care led to a favorable outcome. The patient showed progressive improvement, allowing for successful weaning from NIV and eventual discharge.

Scrub typhus can present with severe multilobar pneumonia and pleural effusion, mimicking bacterial pneumonia, leading to delayed diagnosis and treatment. Early suspicion, timely doxycycline initiation, and respiratory support are crucial for favorable outcomes. Clinicians should consider scrub typhus in atypical pneumonia cases, especially in endemic areas, even in the absence of eschar. Persistent fever and non-resolving pneumonia despite broad-spectrum antibiotics warrant an expanded infectious workup. Further studies should investigate biomarkers for early differentiation of scrub typhus pneumonia, optimal timing for initiating combination therapy, and the long-term pulmonary sequelae in severe cases requiring intensive care.

## Conclusions

Our case underscores the importance of considering scrub typhus as a differential diagnosis in patients with severe multilobar pneumonia, especially in endemic regions. The delayed response to empirical antibiotics should prompt an extended infectious workup to identify atypical pathogens. Early recognition and initiation of doxycycline therapy can significantly improve outcomes, even in cases with severe respiratory involvement. Given the rising incidence of scrub typhus and its potential for severe complications, heightened clinical awareness and timely intervention remain crucial.
